# Exploring the role of community pharmacists in addressing obesity: a Saudi Arabian perspective

**DOI:** 10.3389/fpubh.2025.1503260

**Published:** 2025-03-10

**Authors:** Othman AlOmeir, Mansour Almuqbil, Hanaa Ali Alhabshi, Maha Mahrab Saiel Alenazy, Saleha Mafareh Al-Jaro Masaod Hagwi, Walaa F. Alsanie, Abdulhakeem S. Alamri, Majid Alhomrani, Amal F. Alshammary, Rafiulla Gilkaramenthi, Syed Mohammed Basheeruddin Asdaq

**Affiliations:** ^1^Department of Clinical Pharmacy, College of Pharmacy, Shaqra University, Shaqra, Saudi Arabia; ^2^Department of Clinical Pharmacy, College of Pharmacy, King Saud University, Riyadh, Saudi Arabia; ^3^Department of Pharmacy Practice, College of Pharmacy, AlMaarefa University, Dariyah, Riyadh, Saudi Arabia; ^4^Department of Clinical Laboratory Sciences, The Faculty of Applied Medical Sciences, Taif University, Taif, Saudi Arabia; ^5^Research Center for Health Sciences, Deanship of Graduate Studies and Scientific Research, Taif University, Taif, Saudi Arabia; ^6^Department of Clinical Laboratory Sciences, College of Applied Medical Sciences, King Saud University, Riyadh, Saudi Arabia; ^7^Department of Emergency Medical Services, College of Applied Sciences, AlMaarefa University, Diriyah, Riyadh, Saudi Arabia; ^8^Research Center, Deanship of Scientific Research and Post-Graduate Studies, AlMaarefa University, Dariyah, Riyadh, Saudi Arabia

**Keywords:** belief, barriers, community pharmacist, obesity, practice, weight management

## Abstract

**Introduction:**

Obesity has emerged as a significant public health concern in Saudi Arabia, with rising prevalence rates contributing to an increased risk of chronic diseases such as diabetes and cardiovascular disorders. Community pharmacists, as accessible healthcare providers, hold the potential to play a critical role in weight management. This study aimed to explore the beliefs, practices, and barriers encountered by community pharmacists in Saudi Arabia regarding weight management.

**Methods:**

A cross-sectional survey was conducted among community pharmacists to gather data on their perceptions, practices, and barriers related to obesity management. The data obtained were subjected to descriptive and inferential analysis using a multinomial regression model with the help of SPSS-IBM 2025.

**Results:**

Findings revealed that while a significant majority (73%) recognize obesity as a pressing health issue, only 31% reported receiving formal education on weight management, which impedes their ability to provide effective counseling. The demographic profile of respondents showed a predominance of male pharmacists (91%) aged between 31 and 40 years, which reflects broader societal trends in healthcare professions in the region. Furthermore, barriers such as inadequate staffing (39%), lack of private consultation spaces (37%), and the necessity for additional payment for weight management services (49%) were identified, underscoring the need for targeted support. Pharmacists showed a strong dedication to helping patients adopt healthier lifestyles. Specifically, 76% of pharmacists provided advice on following low-calorie diets, and 83% encouraged patients to increase their physical activity. However, only 33% regularly dispensed weight loss products, indicating a gap in practice.

**Discussion:**

Enhanced education and supportive policies are crucial for pharmacists in obesity management. Future research should focus on developing tailored training programs to fill the knowledge gaps and explore financial incentives to optimize pharmacists’ roles in public health initiatives aimed at combating obesity and chronic diseases in Saudi Arabia. By addressing these barriers, community pharmacists can significantly contribute to obesity management and improve health outcomes in their communities.

## Introduction

Over the past 30 years, the global prevalence of obesity has surged significantly. More than 2.5 billion individuals worldwide are considered overweight, and of these, over 890 million are classified as obese, which has led to its recognition as a global pandemic (1). Obesity is closely associated with an increased risk of morbidity and mortality. It is a major contributing factor to the development of various non-communicable diseases, including type 2 diabetes, stroke, coronary artery disease, dyslipidemia, hypertension, respiratory conditions, and cancers (2). In addition to its physiological consequences, obesity has also been linked to a heightened risk of numerous psychological disorders, such as depression, low self-esteem, eating disorders, anxiety, negative body image, and a diminished quality of life (3). As a result, the ongoing rise in obesity is acknowledged as a significant public health challenge that threatens the health advancements made in many countries. Therefore, implementing evidence-based weight management strategies is crucial to addressing the increasing obesity rates (4).

Changes in lifestyle and growing affluence have particularly contributed to the expanding obesity epidemic in developing nations like Saudi Arabia. The latest research indicates that 24.7% of the population in Saudi Arabia has a body mass index (BMI) exceeding 30 kg/m^2^ (5). Studies have shown that as living standards have improved in Saudi Arabia, there has been an increase in sedentary behaviors and a greater consumption of processed foods and sugary beverages, leading to high obesity rates (6–9). According to a recent study, 35% of men and 22% of women in Saudi Arabia are obese, while 28% of men and 44% of women are overweight (10). Consequently, managing obesity has become a central focus of the Ministry of Health’s (MOH) guidelines in Saudi Arabia. These guidelines aim to establish a comprehensive training program for healthcare professionals, including family medicine consultants and clinical pharmacists, to enhance the prevention, early detection, and management of obesity (11).

While it is well established that physician recommendations significantly impact weight control, several barriers, including time constraints, limited access to lifestyle modification resources, and inadequate reimbursement, create challenges in the patient-physician relationship (12). There is growing evidence that a multidisciplinary approach, combining various healthcare specializations and expertise, is likely to be the most effective (13). Community pharmacists play a crucial role among these healthcare professionals, often serving as the first point of contact for patients within the healthcare system. They are trusted, easily accessible, and engage regularly with patients due to prescription dispensing routines (14). Situated in the heart of communities, pharmacists can contribute to a range of health promotion initiatives, including the prevention of chronic conditions such as diabetes, osteoporosis, hyperlipidemia, and hypercholesterolemia (15–20). The American Society of Health-System Pharmacists advocates for pharmacists to assist obese patients in managing lifestyle modifications (21). Pharmacists receive extensive training on the causes, risk factors, management, and treatment of obesity, making them well-qualified to provide counseling on lifestyle interventions (22). A recent scoping review concluded that weight management interventions led by community pharmacies resulted in modest, but clinically significant, weight loss, which helped improve surrogate markers of cardiovascular disease (23). However, most of the studies included in this review were conducted in developed nations (24–26), with limited data from developing countries, particularly those in the Eastern Mediterranean Region (EMR), where the rising obesity rates are alarming (17, 27).

Several weight-control products are available in Saudi Arabia, including prescription-only medication orlistat (28). On the other hand, products such as herbal teas and dietary supplements can be purchased over the counter (OTC). This study aims to explore the role of community pharmacists in Saudi Arabia in the prevention and treatment of overweight and obesity, with a specific focus on their perceptions of their responsibilities in weight management, as well as the current practices, services, and knowledge they provide. Additionally, the study seeks to identify the challenges that community pharmacists encounter in delivering optimal weight management services.

## Materials and methods

### Study design

This observational cross-sectional study was conducted in Saudi Arabia between September and November 2023. Stratified random sampling was employed for data collection, with stratification based on the five regions of Riyadh city. Within each stratum (governorate), pharmacies were randomly selected, and the number of pharmacies chosen from each stratum was proportional to the total number in that area. Participants received a validated, pre-tested questionnaire via social media and were asked to provide their responses through self-administration. Licensed community pharmacists from the selected pharmacies, with at least 6 months of experience, were eligible for participation. The sample size for this study was calculated to be 377, based on a 5% margin of error, a 95% confidence level, and an estimated population of 2000 pharmacies in the Riyadh region, using an online sample size calculator.^1^ However, due to practical constraints, data was collected from 331 participants. While the final sample size was smaller than the calculated target, it still provides valuable insights. This limitation is acknowledged in the study.

### Development and validation of questionnaire

The questionnaire was developed using published literature (29, 30), aligning with the study’s objectives. The questionnaire comprised four sections:

1. Sociodemographic Information: This section gathered data on gender, age, education level, pharmacy experience, nationality, pharmacy location, age of pharmacy outlet, and education/training of pharmacists in weight management.
2. Beliefs on Weight Management: Eight questions explored community pharmacists’ beliefs on weight management, covering topics like obesity as a growing issue in Saudi Arabia, the role of pharmacists in weight management, the sale of weight-loss products, continuous education needs, potential abuse of such products, and the influence of media and companies.
3. Pharmacist Practices in Weight Management: This section included 10 questions regarding practices such as dispensing weight-loss products, counseling, advising on diet and physical activity, checking interactions, and assessing side effects.
4. Barriers Faced in Weight Management: Seven questions examined challenges such as limited time, staff, space, equipment, and knowledge, as well as lack of interest and absence of service charges.

The questionnaire was translated into Arabic using a back-translation process to ensure linguistic and conceptual equivalence. To validate the newly developed questionnaire, we conducted content, construct validation, and assessed facial validity through a pilot study.

Content Validity: This was established through expert review. A panel of six experts from the fields of pharmacology, community medicine, and community pharmacy assessed the relevance, clarity, and comprehensiveness of the questionnaire items. Based on their feedback, revisions were made to ensure the items accurately represented the intended construct.

Construct Validity: Factor analysis was performed to identify the underlying structure of the questionnaire items related to pharmacists’ beliefs, practices, and barriers in addressing obesity and weight management in Saudi Arabia (31, 32). The analysis used Principal Component Analysis (PCA) with Oblimin rotation, as we expected the factors to be correlated. The Kaiser-Meyer-Olkin (KMO) measure of sampling adequacy was 0.778, indicating that the data were suitable for factor analysis (values above 0.7 are considered acceptable). Bartlett’s Test of Sphericity was significant (χ^2^ = 2660.935, *p* < 0.001), confirming that the correlation matrix was appropriate for factor analysis. The analysis identified eight components with eigenvalues greater than 1, explaining 66.186% of the total variance. The scree plot and eigenvalues supported the retention of eight components, as the slope of the plot leveled off after the eighth component. The communalities for all items ranged from 0.487 to 0.778, suggesting that a substantial proportion of the variance in each item was explained by the extracted components. Internal consistency was assessed using Cronbach’s alpha, with all components demonstrating acceptable reliability (*α* > 0.7). The factor analysis provided a robust and interpretable structure for the questionnaire, revealing eight key components that reflect pharmacists’ perceptions, practices, and barriers in addressing obesity and weight management.

Internal Consistency: The overall internal consistency of the questionnaire was assessed using Cronbach’s alpha, which yielded a value of 0.714, indicating satisfactory reliability.

Facial Validity: A pilot study with a sample of 20 participants from the target population was conducted to test the clarity and feasibility of the questionnaire. Feedback from the pilot study highlighted areas for improvement, such as unclear wording and issues with the response scale. Based on this feedback, necessary revisions were made to enhance the questionnaire’s clarity, conciseness, and relevance to the target audience.

### Data collection

Research team members approached pharmacists in the selected study areas, inviting them to participate. Participants were informed of the study’s objectives, methods, risks, voluntary nature, and the confidentiality of their responses. They were assured that they could withdraw from the study at any time without facing any consequences. Participants self-administered the bilingual questionnaire, with a research team member present to clarify any misunderstandings.

### Statistical methods

Descriptive statistics were used to present the sociodemographic characteristics in Table 1. Data from sections 2, 3, and 4 were analyzed using percentages and frequencies, with Likert-scale responses ranging from strong agreement to strong disagreement. Overall ratings for each section were averaged to assess cohort responses, applying ratings from 5 (strong agreement) to 1 (strong disagreement).

**Table 1:** Sociodemographic characteristics of the participants.

Characteristics	Variables	Frequency	Percentage
Gender	Male	302	91
	Female	29	9
Age	20–30	96	29
	31–40	195	59
	41–50	35	11
	>50	5	2
Education	Diploma	6	2
	BSC	270	82
	Pharm.D	53	16
	Postgraduate	2	1
	PhD	0	0
Experience (years)	<1	10	3
	1–3	54	16
	4–6	56	17
	7–10	83	25
	>10	128	39
Nationality	Saudi	63	19
	Non-Saudi	268	81
Location	North	67	20
	South	65	20
	East	69	21
	West	65	20
	Central	65	20
Age of pharmacy outlet	1–3 years	57	17
	4–10 years	93	28
	>10 years	181	55
Weight management Training	Yes	126	38
	No	205	62
Weight management education	Yes	104	31
	No	227	69

The total score for each domain (belief, practice, and barriers) was calculated by summing the ratings for all items within the domain for each participant, then dividing by the number of items in that domain to obtain an average score per participant. Specifically, the belief domain included 8 items, the practice domain had 10 items, and the barriers domain had 7 items. These domain averages were then classified into categories: belief was categorized as positive (≥4.27) vs. negative (<4.27), practice as good (≥4.21) vs. poor (<4.21), and barriers as high (≥3.38) vs. low (<3.38).

Comparisons of demographic variables with individual items were performed using the Chi-Square test. Multinomial regression analysis was used to identify factors influencing pharmacists’ practices and barriers to effective weight management. Odds ratios, 95% confidence intervals, and *p*-values were calculated, with a significance threshold set at *p* < 0.05. All statistical analyses were conducted using SPSS-IBM 2025.

## Results

### Sociodemographic features of participants

Table 1 summarizes the demographic and professional characteristics of the study participants. A total of 331 individuals participated, with a predominant male representation (91%) compared to females (9%). Age distribution indicates that most participants are aged 31–40 years (59%), followed by those aged 20–30 years (29%). Educational qualifications reveal that the majority hold a Bachelor of Science (82%), while 16% possess a Pharm.D. degree. Experience levels vary, with 39% having over 10 years of experience in the field. Notably, 81% of participants are non-Saudi, and there is a relatively even geographical distribution across different regions. Regarding weight management training, 62% reported not receiving any, and 69% indicated a lack of formal education on the subject.

### Belief of community pharmacist on weight management

Table 2 reflects survey responses on obesity, pharmacists’ involvement in weight management, and the regulation and marketing of weight loss products in Saudi Arabia, incorporating both frequency and mean values. The high mean scores for most items indicate strong agreement. For instance, the question of obesity is a growing issue has a mean of 4.683, reinforcing that 73% strongly agree. Similarly, pharmacists’ role in weight management (mean 4.595) and the need for weight loss products to be sold only in pharmacies (mean 4.511) receive strong support. Continuous education in weight management for pharmacists also shows strong agreement with a mean of 4.532. Although 52% believe patients may misuse weight loss products, the mean of 4.266 indicates a less unanimous response. The lowest means (3.148) relates to the belief that herbal weight loss products are well-regulated, showing significant division. The media’s role in educating patients have a moderate mean of 4.139, suggesting mixed but generally positive views.

**Table 2:** Belief of community pharmacists on weight management (*n* = 331).

Number	Item	Strongly agree	Agree	No comment	Disagree	Strongly disagree	Mean
		Frequency (percentage)					
1	Do you believe obesity is becoming an increasing issue in Saudi Arabia?	242 (73)	78 (24)	6 (2)	5 (2)	0 (0)	4.683
2	Do you think pharmacists have a role in weight management?	223 (67)	89 (27)	12 (4)	7 (2)	0 (0)	4.595
3	Should weight loss products only be available for sale in pharmacies?	219 (66)	81 (24)	14 (4)	15 (5)	2 (1)	4.511
4	Should continuing education for pharmacists include weight management and related training?	211 (64)	94 (28)	17 (5)	9 (3)	0 (0)	4.532
5	In your opinion, are patients misusing weight loss products?	172 (52)	103 (31)	29 (9)	26 (8)	1 (0)	4.266
6	Do you think companies that market weight loss products are making misleading claims?	199 (60)	58 (18)	57 (17)	17 (5)	0 (0)	4.326
7	Do you believe that herbal weight loss products are properly regulated?	64 (19)	106 (32)	35 (11)	67 (20)	59 (18)	3.148
8	Do you think the media and advertisements are contributing positively to educating consumers about weight loss products and weight management?	171 (52)	90 (27)	23 (7)	39 (12)	8 (2)	4.139

### Practice of community pharmacists on weight management

Table 3 presents the practices of community pharmacists regarding weight management, based on responses from 331 participants. The highest mean values are seen for advising patients to increase physical activity (mean 4.746), check for drug or food interactions (mean 4.701), and recommend a low-calorie diet (mean 4.686), reflecting a strong commitment to promoting healthy lifestyle changes. Additionally, most pharmacists (77%) strongly agree they counsel patients on the safe and effective use of weight loss products, supported by a mean of 4.634. Although 44% of pharmacists dispense weight loss products, the mean score of 3.710 suggests varying practices. Interestingly, 41% refer patients to a doctor before dispensing weight loss products, with a mean of 3.955, while 44% ask patients about side effects (mean of 3.937). A notable division is seen in providing BMI calculations, with a lower mean of 3.009, indicating inconsistent practices in this area.

**Table 3:** Practice of community pharmacists on weight management (*n* = 331).

Number	Item	Strongly agree	Agree	No comment	Disagree	Strongly disagree	Mean
		Frequency (percentage)					
1	Do you offer weight loss products for sale at your pharmacy?	109 (33)	83 (25)	91 (28)	30 (9)	18 (5)	3.710
2	Do your patients frequently inquire about weight loss products?	145 (44)	129 (39)	47 (14)	10 (3)	0 (0)	4.236
3	Do you refer patients to a medical doctor before dispensing weight loss products?	137 (41)	65 (23)	89 (27)	24 (7)	5 (2)	3.955
4	Do you provide counseling to patients on the safe and effective use of weight management products?	254 (77)	41 (12)	30 (10)	4 (1)	2 (1)	4.634
5	Do you check for potential drug or food interactions before dispensing weight loss products?	268 (81)	36 (11)	20 (6)	5 (2)	2 (1)	4.701
6	Do you advise patients to follow a low-calorie diet while using weight management products?	251 (76)	57 (17)	22 (7)	1 (0)	0 (0)	4.686
7	Do you recommend increased physical activity to patients using weight loss products?	275 (83)	33 (10)	18 (5)	5 (2)	0 (0)	4.746
8	Do you advise patients to increase their intake of soluble fiber for better weight management?	232 (70)	57 (17)	39 (12)	2 (1)	1 (0)	4.562
9	Do you offer BMI calculations for your patients as part of weight management counseling?	100 (30)	43 (13)	44 (13)	48 (15)	96 (29)	3.009
10	Do you follow up with patients to inquire about any side effects or adverse reactions after taking weight loss products?	144 (44)	74 (22)	67 (20)	40 (12)	6 (2)	3.937

### Barriers faced by community pharmacists to weight management

Table 4 highlights the barriers community pharmacists face in providing weight management services. A significant portion of respondents (39%) strongly agree that insufficient staff is a major barrier, with a mean of 4.024, indicating this is the most prominent challenge. A lack of space for private consultations also poses a significant issue, with 37% strongly agreeing (mean 3.807). The need for additional payment to offer these services was another notable barrier, with nearly half (49%) strongly agreeing, reflected by a means of 4.069. Conversely, fewer pharmacists felt a lack of knowledge (mean 2.456) or interest (mean 2.236) were significant barriers, as most respondents disagreed with these statements. Lack of time (mean 3.607) and relevant equipment (mean 3.465) were moderate concerns, reflecting a need for better resources and support for pharmacists to actively engage in weight management services.

**Table 4:** Barriers to weight management (*n* = 331).

Number	Item	Strongly agree	Agree	No comment	Disagree	Strongly disagree	Mean
		Frequency (percentage)					
1	I do not have sufficient time to offer weight management services.	104 (31)	99 (30)	34 (10)	82 (25)	12 (4)	3.607
2	I lack enough staff to provide weight management services.	128 (39)	130 (39)	34 (10)	31 (9)	8 (2)	4.024
3	I do not have adequate space for a private consultation area to deliver weight management services.	121 (37)	101 (31)	36 (11)	70 (21)	3 (1)	3.807
4	I do not have the necessary equipment (e.g., weighing scale) to offer weight management services.	103 (31)	82 (25)	22 (7)	114 (34)	10 (3)	3.465
5	I would require additional compensation to provide weight management services.	162 (49)	85 (26)	42 (13)	29 (9)	13 (4)	4.069
6	I do not possess enough knowledge to offer weight management services.	47 (14)	20 (6)	31 (9)	172 (52)	61 (18)	2.456
7	I have no interest in providing weight management services.	34 (10)	12 (4)	21 (6)	195 (59)	69 (21)	2.236

### Comparison of sociodemographic characteristics with beliefs of the community pharmacist on weight management

Table 5 presents a comparative analysis of the beliefs of community pharmacists regarding weight management based on various sociodemographic characteristics of 331 respondents. The table categorizes responses by gender, age, education, experience, nationality, location, pharmacy store lifespan, weight management training, and weight management education.

**Table 5:** Comparison of sociodemographic characteristics with beliefs of the community pharmacist on weight management.

Characteristics	Obesity growing problem	Pharmacist role in wt mgt	Products’ sale in pharmacies	CPE on weight mgt	Abuse by patient	Comp false promise	Herbal regulated	media and adv wt. mgt
Gender								
Male	94	92	91	91	85	87	63	83
Female	91	89	83	82	86	82	62	79
*p value	0.098	0.001	0.000	0.000	0.017	0.218	0.887	0.248
Age (years)								
20–30	93	93	92	92	84	81	66	79
31–40	94	92	91	90	85	90	63	85
41–50	94	88	85	87	94	81	58	89
>50	88	88	80	92	76	88	36	56
*p value	0.019	0.433	0.000	0.228	0.194	0.005	0.021	0.000
Education								
Diploma	87	80	83	77	87	93	57	83
BSC	93	92	89	90	85	85	62	82
Pharm.D	97	94	95	93	88	92	67	85
Postgraduate	90	100	100	100	90	70	70	90
*p value	0.131	0.010	0.016	0.010	0.000	0.056	0.208	0.132
Experience (Years)								
<1	96	92	94	90	84	84	76	94
1–3	97	92	90	91	88	88	70	82
4–6	89	89	87	90	85	80	58	73
7–10	93	93	93	91	88	86	58	87
>10	94	92	89	91	83	90	65	84
*p value	0.009	0.702	0.434	0.352	0.252	0.086	0.039	0.003
Nationality								
Saudi	97	91	93	91	88	90	63	83
Non-Saudi	93	92	90	91	85	86	63	83
*p value	0.149	0.100	0.006	0.369	0.013	0.097	0.006	0.101
Location								
North	94	91	90	90	83	89	78	82
South	89	89	86	88	86	82	52	77
East	99	94	97	91	96	92	51	94
West	90	92	86	91	78	78	62	78
Central	96	93	93	94	84	92	71	82
*p value	0.000	0.002	0.000	0.000	0.000	0.000	0.000	0.000
Life of pharmacy store								
1–3 years	91	89	84	88	82	77	62	79
4–10 years	92	91	89	90	84	85	68	83
>10 years	95	93	93	92	87	90	61	84
*p value	0.050	0.383	0.001	0.112	0.141	0.000	0.000	0.447
Weight Mgt training								
Yes	92	93	92	93	88	83	57	89
No	95	91	89	89	84	89	67	79
*p value	0.384	0.012	0.001	0.005	0.000	0.030	0.000	0.000
Weight Mgt education								
Yes	92	92	90	93	84	80	63	83
No	94	92	90	90	89	90	63	83
*p value	0.068	0.223	0.002	0.097	0.000	0.000	0.000	0.01

*Pearson chi-square test.

The findings reveal that a higher percentage of male pharmacists (94%) believe obesity is a growing problem compared to females (91%), with significant differences in perceptions of the pharmacist’s role in weight management (*p* = 0.001). Age also influences beliefs, particularly in the perception of the need for continuous education (CPE) on weight management, where younger pharmacists (20–30 years) show higher agreement (92%).

Education level correlates positively with beliefs, particularly among Pharm. D graduates, who express stronger agreement across various items compared to those with diplomas. Experience impacts beliefs about the abuse of weight loss products, with less experienced pharmacists (<1 year) more likely to perceive abuse as an issue (84%).

Geographical location significantly affects beliefs, especially regarding media and advertisements’ roles in weight management education, with pharmacists in the Eastern region showing the highest agreement (99%). Training and education in weight management also positively influence beliefs, highlighting the importance of professional development in shaping pharmacists’ perceptions and practices related to weight management. Overall, these results underscore the interplay between sociodemographic factors and pharmacists’ beliefs, which can inform targeted interventions to enhance weight management services in community pharmacies.

### Comparison of sociodemographic characteristics with practices of the community pharmacist on weight management

Table 6 compares the sociodemographic characteristics of community pharmacists with their practices related to weight management. Gender differences show that male pharmacists tend to be more proactive in checking for drug or food interactions (*p* = 0.035), referring patients to doctors (*p* = 0.022), and advising low-calorie diets (*p* = 0.003), while female pharmacists perform similarly in most other areas.

**Table 6:** Comparison of sociodemographic characteristics with practices of the community pharmacist on weight management.

Characteristics	Dispense wt. products	Patients ask for wt. loss products	Refer to doctor before dispensing	Counsel on product use	Check drug/food interactions	Advise low-calorie diet	Advise physical activity	Advise soluble fiber	Provide BMI calculation	Ask about side effects
Gender										
Male	75	85	80	93	94	94	95	92	60	79
Female	68	83	72	90	90	86	90	86	59	77
*p value	0.34	0.486	0.022	0.521	0.035	0.003	0.020	0.010	0.872	0.085
Age (years)										
20–30	74	83	78	94	93	94	94	90	63	79
31–40	73	85	82	93	95	94	96	93	57	77
41–50	79	85	72	88	91	90	93	87	75	89
>50	92	92	52	96	96	96	92	92	24	76
*p value	0.398	0.681	0.000	0.583	0.456	0.222	0.788	0.272	0.00	0.002
Education										
Diploma	77	93	80	80	83	73	70	73	60	70
BSC	74	85	79	93	94	94	95	91	60	80
Pharm.D	76	82	82	94	96	96	97	94	62	74
Postgraduate	90	90	60	100	100	90	90	80	80	90
*p value	0.299	0.805	0.064	0.261	0.519	0.004	0.000	0.000	0.918	0.170
Experience (Years)										
<1	80	76	88	94	98	94	92	88	62	62
1–3	79	87	84	93	93	95	97	93	66	81
4–6	74	79	71	89	87	89	90	88	70	82
7–10	72	85	77	92	95	94	94	91	65	82
>10	73	86	81	94	97	95	97	93	50	75
*p value	0.794	0.103	0.007	0.005	0.005	0.099	0.011	0.000	0.006	0.000
Nationality										
Saudi	74	85	81	93	92	92	94	91	64	73
Non-Saudi	74	85	79	93	94	94	95	91	59	80
*p value	0.539	0.416	0.352	0.692	0.159	0.378	0.284	0.215	0.610	0.171
Location										
North	62	82	78	90	96	98	97	93	37	62
South	80	85	74	93	91	87	89	90	70	88
East	80	85	84	93	94	96	96	95	86	92
West	75	82	73	93	94	94	95	87	56	81
Central	73	90	87	94	96	94	97	91	51	70
*p value	0.000	0.039	0.000	0.556	0.059	0.001	0.050	0.001	0.000	0.000
Life of pharmacy store										
1–3 years	68	80	75	92	89	88	91	85	68	86
4–10 years	72	82	78	92	94	95	96	91	61	78
>10 years	77	88	81	93	95	95	96	93	57	77
*p value	0.144	0.000	0.034	0.023	0.131	0.001	0.048	0.000	0.000	0.061
Weight Mgt training										
Yes	80	85	81	92	94	92	93	91	77	90
No	71	85	78	93	94	95	96	92	50	72
*p value	0.007	0.210	0.324	0.599	0.348	0.075	0.050	0.205	0.000	0.000
Weight Mgt education										
Yes	80	85	77	93	93	90	91	88	75	89
No	71	84	80	93	94	95	97	93	54	74
*p value	0.001	0.361	0.288	0.117	0.259	0.002	0.000	0.004	0.000	0.000

*Pearson chi-square test.

Age influences weight management practices, with pharmacists over 50 years being less likely to refer patients to doctors (*p* = 0.000) or provide BMI calculations (*p* = 0.00) compared to younger age groups. Education significantly impacts practices, with postgraduate pharmacists showing better adherence to counseling, checking interactions, and calculating BMI compared to those with diplomas (*p* < 0.05).

Experience is another key factor, as pharmacists with more than 10 years of experience excel in checking drug interactions, providing BMI calculations, and asking about side effects (*p* < 0.05). In contrast, less experienced pharmacists show lower performance in these areas. Geographical location also plays a role, with pharmacists from the central and eastern regions performing better in practices like BMI calculation and checking interactions (*p* < 0.05).

Weight management training and education significantly improve practices, especially in providing BMI calculations and asking about side effects, where trained pharmacists show higher engagement (*p* = 0.000).

### Comparison of sociodemographic characteristics with barriers faced by community pharmacists in weight management

Table 7 highlights the barriers faced by community pharmacists in weight management, with a focus on sociodemographic characteristics. Gender-wise, female pharmacists reported higher barriers in areas like knowledge (*p* = 0.010) and interest (*p* = 0.041), compared to their male counterparts. Age also played a role, with pharmacists over 50 years experiencing more challenges across most categories, including time (*p* = 0.005), staff (*p* = 0.000), and space for consultation (*p* = 0.000).

**Table 7:** Comparison of sociodemographic characteristics with barriers faced by community pharmacists in weight management.

Characteristics	No time	No staff	No space for consultation	No equipment	No additional pay	No knowledge	No interest
Gender							
Male	72	80	75	69	81	48	44
Female	74	83	86	75	83	64	57
*p value	0.988	0.716	0.082	0.446	0.937	0.010	0.041
Age (years)							
20–30	68	75	73	67	77	49	42
31–40	73	83	77	70	84	47	44
41–50	75	80	80	71	81	59	51
>50	84	84	84	84	84	52	84
*p value	0.005	0.000	0.000	0.040	0.003	0.004	0.000
Education							
Diploma	100	100	93	93	83	77	80
BSC	71	80	75	68	80	49	44
Pharm.D	77	83	80	72	91	48	46
Postgraduate	40	60	60	50	60	30	30
*p value	0.002	0.038	0.083	0.001	0.083	0.013	0.009
Experience (Years)							
<1	76	76	80	66	84	54	50
1–3	75	83	79	70	85	56	49
4–6	70	73	75	71	74	53	49
7–10	74	82	79	73	83	49	43
>10	71	82	73	66	82	44	42
*p value	0.686	0.057	0.194	0.108	0.421	0.291	0.258
Nationality							
Saudi	76	80	79	73	87	56	49
Non-Saudi	71	81	75	69	80	48	44
*p value	0.063	0.339	0.096	0.080	0.152	0.213	0.237
Location							
North	62	81	75	67	84	42	39
South	81	82	82	81	81	59	60
East	87	89	88	76	93	54	46
West	60	70	65	63	67	47	39
Central	70	80	70	59	82	44	40
*p value	0.000	0.000	0.000	0.000	0.000	0.000	0.000
Life of pharmacy store							
1–3 years	69	75	75	67	67	58	49
4–10 years	73	78	73	69	80	49	45
>10 years	72	83	78	70	87	46	43
*p value	0.025	0.002	0.006	0.085	0.000	0.001	0.004
Weight Mgt training							
Yes	74	81	77	73	76	50	47
No	71	80	76	67	84	48	44
*p value	0.012	0.001	0.002	0.000	0.000	0.000	0.000
Weight Mgt education							
Yes	70	78	73	70	76	53	51
No	73	82	78	69	84	47	42
*p value	0.001	0.000	0.000	0.000	0.000	0.004	0.000

*Pearson chi-square test.

Education level significantly influenced these barriers, as diploma holders faced the most obstacles, particularly in terms of time, equipment, and knowledge (*p* < 0.05). Conversely, postgraduate pharmacists reported fewer challenges, with significantly lower percentages in all areas except additional pay. Experience also had an impact, though less pronounced; pharmacists with less experience (<1 year) reported slightly higher barriers compared to those with over 10 years in the field.

Nationality did not show significant differences in most categories. However, regional variations were notable, with pharmacists from the east and south reporting higher challenges in staffing, space, and equipment (*p* < 0.05). Additionally, longer-established pharmacy stores (over 10 years) tended to face fewer issues with pay and knowledge (*p* = 0.000).

Weight management training and education reduced several barriers, especially regarding staff, equipment, and knowledge (*p* < 0.05).

### Factors that influence the belief of community pharmacists on weight management

Supplementary Table 1 presents the results of a multinomial regression analysis examining factors that influence community pharmacists’ beliefs about weight management. The reference category for belief is set as “Negative belief with an average score of <4.27.”

Gender: Male pharmacists are more likely to have positive beliefs about weight management, with an odds ratio (OR) of 2.549 compared to females (*p* = 0.065), though this is not statistically significant.

Age: Pharmacists aged 31–40 are more likely to have positive beliefs, with an OR of 11.315, though not statistically significant (*p* = 0.058). The 41–50 age group also shows increased odds (OR = 7.917), but the *p*-value (0.108) indicates no strong evidence of association.

Education: Educational attainment does not significantly influence beliefs, as none of the education categories (Diploma, BSc, Pharm.D) show significant *p*-values. The odds ratios vary widely, with Pharm. D pharmacists showing slightly higher odds (OR = 1.549) compared to postgraduate pharmacists (reference group).

Experience: Years of experience do not strongly affect beliefs, with no significant p-values across the categories. Those with 4–6 years of experience are less likely to have positive beliefs (OR = 0.501), though the result is not significant (*p* = 0.138).

Nationality: Saudi pharmacists are less likely to have positive beliefs (OR = 0.800), though this is not statistically significant (*p* = 0.676).

Location: Pharmacists in the North (*p* = 0.013), South (*p* = 0.036), and West (*p* = 0.000) regions are significantly less likely to have positive beliefs about weight management compared to those in the Central region.

Life of Pharmacy Store: Pharmacists working in stores that have been open for 1–3 years show significantly lower odds of having positive beliefs (OR = 0.253, *p* = 0.001) compared to those in stores older than 10 years.

Weight Management Training & Education: Neither training nor education in weight management significantly influences pharmacists’ beliefs, with *p*-values of 0.112 and 0.367, respectively.

In summary, location and the life of the pharmacy store emerge as the most significant factors influencing pharmacists’ beliefs regarding weight management, while factors like gender, education, and experience do not show strong statistical associations.

### Factors that influence the practice of community pharmacist

Supplementary Table 2 presents the multinomial regression analysis of factors influencing the practices of community pharmacists regarding weight management. Here are the key findings:

Gender: Males show a tendency toward better practices, with an odds ratio of 2.867 (*p* = 0.059), although this is marginally non-significant. Females are the reference group.

Age: Pharmacists aged 20–30, 31–40, and 41–50 have significantly improved practices with odds ratios of 9.109 (*p* = 0.009), 9.515 (*p* = 0.007), and 9.295 (*p* = 0.008), respectively. The >50 age group serves as the reference, indicating younger pharmacists practice weight management more effectively.

Education: Education level significantly impacts practices. Those with a Diploma (odds ratio 6.018, *p* = 0.000), BSc (5.415, *p* = 0.000), and Pharm. D (2.226, *p* = 0.000) show higher odds of practicing weight management effectively compared to the postgraduate reference group.

Experience: Pharmacists with less than 1 year of experience show an odds ratio of 8.986 (*p* = 0.034), indicating they may be more engaged in weight management practices compared to those with >10 years of experience. The 1–3 years group is on the edge of significance (*p* = 0.061).

Nationality: Saudi pharmacists have significantly lower odds of poor practices (odds ratio 0.234, *p* = 0.014) compared to non-Saudi pharmacists.

Location: Those in the Eastern region exhibit notably better practices (odds ratio 5.595, *p* = 0.000), whereas no significant differences are observed for other regions.

Life of Pharmacy Store: Pharmacies open for 1–3 years have lower odds of poor practices (odds ratio 0.228, *p* = 0.001), while those established for longer show no significant differences.

Weight Management Training and Education: Training positively influences practices (odds ratio 1.992, *p* = 0.028), whereas education shows a trend toward better practices (odds ratio 1.763, *p* = 0.100), though not statistically significant.

Overall, the analysis reveals that age, education, experience, nationality, location, and training significantly influence the quality of weight management practices among community pharmacists, highlighting the need for targeted training and support initiatives.

### Factors that influence the barriers posed to community pharmacists on weight management

Supplementary Table 3 outlines the multinomial regression analysis assessing the factors associated with barriers experienced by community pharmacists in weight management. The findings highlight several significant predictors:

Gender: The odds ratio for males is 1.314 (*p* = 0.597), indicating no significant difference in barriers faced compared to females, who are the reference group.

Age: Pharmacists aged 20–30 experience significantly higher barriers with an odds ratio of 14.607 (*p* = 0.037), while older age groups (31–40 and 41–50) show no significant differences.

Education: All education levels except for postgraduate significantly affects barriers. Pharmacists with a Diploma (odds ratio 3.951, *p* = 0.000), BSc (3.669, *p* = 0.000), and Pharm. D (6.326, *p* = 0.000) face more barriers compared to postgraduate pharmacists.

Experience: Those with 1–3 years of experience exhibit significantly lower barriers (odds ratio 0.244, *p* = 0.019). Others do not show significant differences compared to those with >10 years of experience.

Nationality: There are no significant differences in barriers faced by Saudi pharmacists (odds ratio 1.438, *p* = 0.497) compared to non-Saudi pharmacists.

Location: Pharmacists in the South (odds ratio 0.358, *p* = 0.016) and East (0.252, *p* = 0.001) regions report fewer barriers, while no significant differences are noted for North, West, or Central locations.

Life of Pharmacy Store: No significant differences in barriers are observed based on how long the pharmacy has been operating.

Weight Management Training: Training does not significantly affect the barriers experienced (odds ratio 0.695, *p* = 0.243).

Weight Management Education: Those with weight management education have significantly lower barriers (odds ratio 2.043, *p* = 0.038).

In conclusion, the analysis identifies age, education level, and location as significant factors influencing the barriers community pharmacists encounter in weight management, emphasizing the importance of targeted education and support to address these challenges.

### Correlation between belief, practice, and barriers in weight management

The correlation matrix presents the relationships between practice, barriers, and beliefs among community pharmacists, with a sample size of 331. The data reveal significant correlations, indicating how these variables interact with one another (Table 8).

**Table 8:** Pearson correlations of outcome variables (belief, practice, and barriers).

		Practice	Barrier	Belief
Practice	Pearson Correlation	1	0.128^*^	0.330^**^
	Sig. (2-tailed)		0.020	0.000
	N	331	331	331
Barrier	Pearson Correlation	0.128^*^	1	0.174^**^
	Sig. (2-tailed)	0.020		0.001
	N	331	331	331
Belief	Pearson Correlation	0.330^**^	0.174^**^	1
	Sig. (2-tailed)	0.000	0.001	
	N	331	331	331

*Correlation is significant at the 0.05 level (2-tailed). **Correlation is significant at the 0.01 level (2-tailed).

Practice and Barrier: There is a positive correlation between practice and barriers (*r* = 0.128, *p* = 0.020), suggesting that as barriers to effective weight management increase, pharmacists’ practices tend to be less aligned with optimal weight management strategies. Although significant, the correlation is relatively weak, indicating that other factors might also contribute to the practices of pharmacists.

Practice and Belief: A stronger positive correlation exists between practice and belief (*r* = 0.330, *p* = 0.000), indicating that pharmacists who engage in effective weight management practices are more likely to hold positive beliefs about their role in this area. This suggests that enhancing practices could positively influence beliefs.

Barrier and Belief: The correlation between barriers and beliefs is also positive but weaker (*r* = 0.174, *p* = 0.001). This implies that higher perceived barriers are associated with less favorable beliefs about weight management, although the impact is not as strong as between practice and belief.

Overall, the analysis indicates that while all three variables are interrelated, the most substantial relationship exists between practice and belief. Addressing the barriers may help improve practices and subsequently enhance pharmacists’ beliefs in their role in weight management. These insights underscore the importance of tackling barriers to optimize both practices and beliefs among community pharmacists.

## Discussion

This study aimed to investigate the beliefs, practices, and barriers faced by community pharmacists in Saudi Arabia regarding weight management, as well as the sociodemographic factors influencing these aspects. The results reveal significant insights into how these professionals perceive and manage weight-related health issues in their communities, which is particularly relevant given the increasing prevalence of obesity in Saudi Arabia.

### Sociodemographic characteristics

The demographic profile of community pharmacists in Saudi Arabia, predominantly male (91%) and within the age range of 31–40 years (59%), is consistent with trends observed in other studies within the region. For instance, Al-Jedai et al. reported a similar male dominance in pharmacy practice, reflecting broader societal trends in healthcare professions within Saudi Arabia (33). However, this contrasts with findings from international studies, such as those conducted in Canada and the United States, where gender representation among pharmacists is more balanced, indicating a potential cultural difference in workforce participation (34). The prevalence of pharmacists holding Bachelor’s degrees (82%) aligns with the findings of Saleh et al. (35), who also noted a significant number of pharmacists lacking postgraduate education (35). This highlights a common challenge in the region, emphasizing the need for further educational opportunities to enhance professional competence in emerging areas like obesity management.

### Beliefs about weight management

The strong acknowledgment (73%) among pharmacists of obesity as a growing health issue mirrors findings from studies in other Middle Eastern countries, such as Kuwait, where pharmacists identified obesity as a primary public health concern (17). This consensus supports the notion that community pharmacists recognize their potential role in addressing this epidemic, as highlighted in international literature that emphasizes the pharmacist’s role as an accessible healthcare provider (36, 37). However, while 67% of Saudi pharmacists believe they have a role in weight management, a study by Hijazi et al. (38) in the Lebanon reported higher engagement levels, with 85% of pharmacists actively participating in obesity management initiatives (38). This discrepancy may point to varying healthcare system dynamics and professional roles in different countries.

The finding that only 31% of pharmacists received formal education on weight management is concerning and aligns with research by Alhazmi et al. (39), which found similar gaps in training among healthcare professionals in Saudi Arabia (39). This lack of education could impede the pharmacists’ confidence and ability to provide effective weight management counseling, echoing the findings of multiple international studies that highlight the importance of structured training programs in enhancing pharmacists’ capabilities in managing chronic diseases (37, 40).

### Practices of community pharmacists in weight management

Regarding practices, the study found that 81% of pharmacists check for drug interactions when dispensing weight loss products. This finding is comparable to a study conducted by Rasheed et al. (41) which reported that pharmacists prioritize patient safety by checking for interactions (41). However, the relatively low rate of 33% dispensing weight loss products regularly raises concerns. In contrast, international studies, such as those from Australia and the United States, show that pharmacists are more actively involved in dispensing and counseling for weight loss products, reflecting a more integrated approach to weight management in those countries (24, 42).

Moreover, the high rate of counseling on lifestyle modifications (e.g., low-calorie diets at 76% and physical activity encouragement at 83%) is consistent with the recommendations from the World Health Organization (WHO), which advocates for comprehensive lifestyle changes as fundamental to obesity management (43). Nevertheless, the lower rate of conducting BMI assessments (30%) indicates a missed opportunity for comprehensive patient assessments, as recommended by public health guidelines. This gap aligns with findings from international research where pharmacists reported similar challenges in routinely performing patient assessments (44).

### Barriers to effective weight management

The perceived barriers to effective weight management services—time constraints (31%), staffing issues (39%), and lack of private consultation spaces (37%)—reflect challenges documented in the literature. For instance, a study in Jordan identified similar barriers hindering pharmacists from fully engaging in public health initiatives (45). In Saudi Arabia, pharmacists often face heavy workloads due to the high demand for pharmaceutical services, leaving limited time for additional responsibilities such as weight management counseling. This aligns with findings from a study in Saudi Arabia, where pharmacists reported that time constraints and understaffing were significant barriers to their involvement in public health programs (46). Similarly, a study in Kuwait found that pharmacists’ engagement in health promotion activities was limited by their primary focus on dispensing medications and managing pharmacy operations (17).

These limitations resonate with global findings that underscore resource constraints as a significant obstacle to optimizing pharmacists’ roles in chronic disease management (47). A study in the Canada highlighted that community pharmacists often struggle to balance their traditional roles with newer responsibilities, such as chronic disease management and preventive care, due to time and staffing limitations (48). These findings underscore the need for systemic changes, such as increased staffing and workflow optimization, to enable pharmacists to take on expanded roles in weight management.

Furthermore, the concern regarding the need for additional payment for weight management services (49%) parallels findings from studies in the United States and Canada, where financial compensation models for pharmacists’ services remain underdeveloped (49). The gap in financial incentives can deter pharmacists from pursuing weight management services, which has been similarly highlighted in research emphasizing the need for appropriate remuneration to encourage pharmacist engagement in public health (50).

The barriers to effective weight management services identified in this study are consistent with findings from both regional and global literature. Addressing these challenges requires a collaborative effort involving policymakers, pharmacy educators, and healthcare stakeholders to create an enabling environment for pharmacists to contribute meaningfully to obesity prevention and management. By addressing these barriers, pharmacists can play a pivotal role in improving public health outcomes related to obesity and chronic disease management.

### Comparison of sociodemographic characteristics with beliefs and practices

The study’s findings regarding gender differences in beliefs about weight management are echoed in the literature, where female pharmacists have been reported to express varying levels of confidence in their roles compared to their male counterparts (51). This finding highlights a cultural aspect that may influence practice behaviors and perceptions among pharmacists in Saudi Arabia, akin to observations in other conservative societies where gender roles are pronounced (52). Additionally, the impact of educational attainment on pharmacists’ beliefs regarding product regulation and media influence is noteworthy. This aligns with previous studies indicating that pharmacists with advanced degrees tend to have greater confidence in their clinical knowledge and are more proactive in their practices (53). However, the lack of significant variation in practices based on experience levels suggests that experiential learning may not be fully utilized within the community pharmacy context, highlighting a need for ongoing professional development that fosters the practical application of knowledge (54).

### Comparison of sociodemographic characteristics with barriers

The study’s findings highlight significant barriers faced by community pharmacists in weight management, with younger pharmacists (ages 20–30) experiencing greater challenges (OR = 14.607, *p* = 0.037), aligning with Wu et al. (55). Additionally, those with lower educational qualifications, particularly diplomas (OR = 3.951, *p* < 0.001), encountered more obstacles compared to postgraduate pharmacists, reflecting Lin et al. (56) findings on the importance of advanced education (56). Geographically, pharmacists in urban areas, especially the Eastern region (OR = 0.252, *p* = 0.001), reported fewer barriers, echoing Hurley-Kim et al. (57). Furthermore, while weight management training did not correlate with barriers (*p* = 0.243), formal education positively influenced confidence (OR = 2.043, *p* = 0.038), as noted by George et al. (58).

### Correlation among beliefs, practices, and barriers

The Pearson correlations presented in Table 8 illustrate significant relationships among belief, practice, and barriers. The correlation between practice and belief (*r* = 0.330, *p* < 0.01) indicates that pharmacists who strongly believe in the importance of weight management are more likely to engage in related practices. This supports findings from a systematic review by Phelan et al. (59), which showed that healthcare providers’ beliefs significantly impact their engagement in weight management activities (59). Conversely, the relationship between barriers and practice (*r* = 0.128, *p* < 0.05) highlights how increased barriers correlate with decreased engagement, suggesting that interventions to reduce barriers may improve practice (60).

### Limitations

While this study provides valuable insights into the beliefs, practices, and barriers of community pharmacists regarding weight management, several limitations must be acknowledged. Firstly, the study relied on self-reported data, which may introduce bias, as pharmacists might overestimate their engagement in weight management practices. Secondly, cross-sectional design limits the ability to establish causality between sociodemographic characteristics and practices. Future longitudinal studies could help elucidate these relationships more clearly.

Additionally, the sample size, although relatively large, was slightly below the originally calculated target of 377, with only 331 participants included. This reduction in sample size may impact on the precision and generalizability of the findings. This limitation should be considered when interpreting the results.

Furthermore, the sample size may not fully represent the diverse pharmacy landscape across all regions of Saudi Arabia. Expanding the geographic scope and including a wider array of pharmacy settings could enhance the generalizability of the findings. Finally, exploring the perspectives of patients regarding their interactions with pharmacists could provide a more comprehensive understanding of the weight management dynamics within the community pharmacy setting.

### Policy implications and recommendations for implementation

The findings of this study highlight the need for targeted interventions and policy reforms to enhance the role of community pharmacists in weight management. To address the barriers identified, such as time constraints, staffing issues, lack of private consultation spaces, and inadequate financial incentives, a multi-faceted approach is required. Below, we outline a detailed roadmap for healthcare authorities, pharmacy institutions, and policymakers to implement these recommendations effectively.

First, the development of targeted training programs is essential to address the knowledge gaps identified in this study. Collaborations between pharmacy schools, professional organizations, and healthcare authorities should be established to design and deliver continuing education programs focused on weight management, nutrition, physical activity, and behavioral counseling. These programs should incorporate evidence-based guidelines and practical tools, such as BMI calculators and dietary assessment tools, to enhance pharmacists’ confidence and competence. Additionally, accredited certification programs in weight management could incentivize participation and ensure standardization of skills across the profession.

Second, financial incentives and reimbursement models must be developed to encourage pharmacists to engage in weight management services. Policymakers should explore reimbursement models that compensate pharmacists for cognitive services, such as weight management counseling and follow-up consultations. Pilot programs, such as pay-for-performance initiatives, could reward pharmacists for achieving measurable patient outcomes, such as weight loss or improved metabolic markers. Furthermore, advocating for the inclusion of pharmacist-provided weight management services in national health insurance schemes would ensure sustainability.

Third, infrastructure development is critical to creating an enabling environment for pharmacists to deliver weight management services effectively. Healthcare authorities should mandate the inclusion of private consultation spaces in community pharmacy design standards to ensure patient privacy and confidentiality. Government grants or subsidies could be provided to pharmacies for the purchase of necessary equipment, such as weighing scales, body composition analyzers, and blood pressure monitors. Additionally, the development of telehealth platforms could facilitate remote consultations and follow-ups, particularly in underserved areas.

Fourth, fostering collaborative care models is essential to improving patient outcomes in weight management. Establishing referral networks between pharmacists, physicians, dietitians, and other healthcare providers would ensure coordinated care for patients with obesity. Shared care protocols should be developed to outline the roles and responsibilities of each healthcare professional in managing obesity and related comorbidities. The use of electronic health records (EHRs) could facilitate seamless communication and information sharing among healthcare providers.

Fifth, public awareness campaigns are needed to increase public awareness of the role of pharmacists in weight management and encourage the utilization of these services. Nationwide campaigns should be launched to educate the public about the benefits of pharmacist-provided weight management services. Social media platforms and community outreach programs could be used to disseminate information and promote healthy lifestyle choices. Partnerships with local schools, workplaces, and community centers could deliver weight management workshops and screenings.

Finally, research and evaluation should be conducted to continuously monitor and evaluate the impact of implemented policies and programs. Longitudinal studies could assess the effectiveness of pharmacist-provided weight management services in improving patient outcomes. Key performance indicators (KPIs), such as patient satisfaction, weight loss, and adherence to treatment plans, should be established to measure the success of interventions. Qualitative research methods could gather feedback from pharmacists and patients on the barriers and facilitators of weight management services.

Overall, the implementation of these recommendations requires a collaborative effort involving healthcare authorities, pharmacy institutions, policymakers, and the community. By addressing the identified barriers and leveraging the potential of pharmacists, Saudi Arabia can develop a robust framework for weight management that improves public health outcomes and reduces the burden of obesity. Future research should focus on evaluating the impact of these interventions and identifying best practices for scaling up successful initiatives.

## Conclusion

Overall, the findings of this study resonate well with existing literature, highlighting the multifaceted barriers to weight management encountered by community pharmacists in Saudi Arabia. By identifying key demographic, educational, and geographic factors influencing these barriers, targeted strategies can be developed to enhance pharmacists’ roles in effectively managing obesity. The results reveal a clear recognition of the obesity epidemic among pharmacists and a willingness to engage, yet significant gaps in education, practical application, and resource availability hinder optimal service delivery. These findings align with both national and international studies, emphasizing the need for continued professional education, supportive policies, and collaborative approaches. Future research should evaluate the effectiveness of tailored training and support programs to address the unique challenges identified in this study, ultimately enhancing community pharmacists’ contributions to public health initiatives targeting obesity and chronic disease management.

## Data Availability

The original contributions presented in the study are included in the article/Supplementary material, further inquiries can be directed to the corresponding author.

## Footnotes

^1^ http://www.raosoft.com/samplesize.html
