# Supplementary material for: Exploring the role of community pharmacists in addressing obesity: a Saudi Arabian perspective
Source: Front Public Health. 2025 Mar 10;13:1503260. doi: 10.3389/fpubh.2025.1503260 (PMC11931123; doi:10.3389/fpubh.2025.1503260)
Supplement: Supplementary file 1 [file Table_1.docx]

**Exploring the Role of Community Pharmacists in Addressing Obesity: A Saudi Arabian Perspective**

**Othman AlOmeir^1^, Mansour Almuqbil^2^, Hanaa Ali Alhabshi^3^,**

**Maha Mahrab Saiel Alenazy^3^, Saleha Mafareh Al-Jaro Masaod Hagwi^3^, Walaa F. Alsanie^4,5^, Abdulhakeem S. Alamri^4,5^, Majid Alhomrani^4,5^, Amal F. Alshammary^6^, Rafiulla Gilkaramenthi^7^, Syed Mohammed Basheeruddin Asdaq^3,8*^**

^1^Department of Clinical Pharmacy, College of Pharmacy, Shaqra University, Shaqra 11961, Saudi Arabia, O.k.alomeir@gmail.com

^2^Department of Clinical Pharmacy, College of Pharmacy, King Saud University, Riyadh 11451, Saudi Arabia, mmetwazi@ksu.edu.sa (M.A.)

^3^Department of Pharmacy Practice, College of Pharmacy, AlMaarefa University, Dariyah, 13713, Riyadh, Saudi Arabia, sasdaq@gmail.com (SMBA); [182220532@student.um.edu.sa](mailto:182220532@student.um.edu.sa) (HAA); [202220514@student.um.edu.sa](mailto:202220514@student.um.edu.sa) (MMSA); [181220324@student.um.edu.sa](mailto:181220324@student.um.edu.sa) (SMAMH)

^4^Department of Clinical Laboratory Sciences, The faculty of Applied Medical Sciences, Taif University, Taif, Saudi Arabia, w.alsanie@tu.edu.sa (WFA); a.alamri@tu.edu.sa (ASA); m.alhomrani@tu.edu.sa (MA)

^5^Research center for health sciences, Deanship of Graduate Studies and Scientific Research, Taif University, Taif 26432, Saudi Arabia

^6^Department of Clinical Laboratory Sciences, College of Applied Medical Sciences, King Saud University, [aalshammary@ksu.edu.sa](mailto:aalshammary@ksu.edu.sa)

^7^Department of Emergency Medical Services, College of Applied Sciences, AlMaarefa University, Diriyah, 13713, Riyadh, Saudi Arabia, [grafi@mcst.edu.sa](mailto:grafi@mcst.edu.sa)

^8^Research Center, Deanship of Scientific Research and Post-Graduate Studies, AlMaarefa University, Dariyah, 13713, Riyadh, Saudi Arabia

*** Correspondence:**Syed Mohammed Basheeruddin Asdaq,

[sasdaq@gmail.com](mailto:sasdaq@gmail.com)

**Supplementary Table 1: Multinomial Regression analysis of factors that** **influence the belief of community pharmacists on weight management.**

| **Characteristics*** | **Significance**  **(*P* value)** | **Odds ratio** | **95% Confidence Interval for Odds ratio** | |
| --- | --- | --- | --- | --- |
|  |  |  | **Lower Bound** | **Upper Bound** |
| **Gender** |  |  |  |  |
| Male | 0.065 | 2.549 | 0.942 | 6.897 |
| Female | Ref | -- | -- | -- |
| **Age (years)** |  |  |  |  |
| 20-30 | 0.187 | 5.805 | 0.425 | 79.319 |
| 31-40 | 0.058 | 11.315 | 0.922 | 138.817 |
| 41-50 | 0.108 | 7.917 | 0.637 | 98.364 |
| >50 | Ref | -- | -- | -- |
| **Education** |  |  |  |  |
| Diploma | 0.508 | 0.163 | 0.001 | 35.057 |
| BSC | 0.758 | 0.471 | 0.004 | 56.965 |
| Pharm.D | 0.861 | 1.549 | 0.012 | 206.430 |
| Postgraduate | Ref | -- | -- | -- |
| **Experience (Years)** |  |  |  |  |
| <1 | 0.993 | 1.008 | 0.162 | 6.263 |
| 1-3 | 0.527 | 1.457 | 0.454 | 4.681 |
| 4-6 | 0.138 | 0.501 | 0.201 | 1.249 |
| 7-10 | 0.607 | 1.195 | 0.606 | 2.358 |
| >10 | Ref | -- | -- | -- |
| **Nationality** |  |  |  |  |
| Saudi | 0.676 | 0.800 | 0.281 | 2.278 |
| Non-Saudi | Ref | -- | -- | -- |
| **Location** |  |  |  |  |
| North | **0.013** | 0.381 | 0.177 | 0.819 |
| South | 0.036 | 0.392 | 0.164 | 0.940 |
| East | 0.771 | 0.884 | 0.385 | 2.029 |
| West | 0.000 | 0.197 | 0.084 | 0.459 |
| Central | Ref | -- | -- | -- |
| **Life of Pharmacy Store** |  |  |  |  |
| 1-3 years | **0.001** | 0.253 | 0.110 | 0.579 |
| 4-10 years | 0.138 | 0.632 | 0.345 | 1.158 |
| >10 years | Ref | -- | -- | -- |
| **Weight Mgt Training** |  |  |  |  |
| Yes | 0.112 | 1.668 | 0.888 | 3.132 |
| No | Ref | -- | -- | -- |
| **Weight Mgt Education** |  |  |  |  |
| Yes | 0.367 | 1.360 | 0.697 | 2.651 |
| No | Ref | -- | -- | -- |

*The reference category for belief is “Negative belief with an average score of <4.27.”

**Supplementary Table 2: Multinomial Regression analysis of factors that influence the practice of community pharmacists on weight management.**

| **Characteristics*** | **Significance**  **(*P* value)** | **Odds ratio** | **95% Confidence Interval for Odds ratio** | |
| --- | --- | --- | --- | --- |
|  |  |  | **Lower Bound** | **Upper Bound** |
| **Gender** |  |  |  |  |
| Male | 0.059 | 2.867 | 0.959 | 8.568 |
| Female | Ref | -- | -- | -- |
| **Age (years)** |  |  |  |  |
| 20-30 | **0.009** | 9.109 | 0.689 | 12.497 |
| 31-40 | **0.007** | 9.515 | 0.816 | 11.893 |
| 41-50 | **0.008** | 9.295 | 0.779 | 11.856 |
| >50 | Ref | -- | -- | -- |
| **Education** |  |  |  |  |
| Diploma | **0.000** | 6.018 | 2.122 | 12.245 |
| BSC | **0.000** | 5.415 | 1.342 | 10.231 |
| Pharm.D | **0.000** | 2.226 | 1.139 | 2.783 |
| Postgraduate | Ref | -- | -- | -- |
| **Experience (Years)** |  |  |  |  |
| <1 | **0.034** | 8.986 | 1.178 | 68.528 |
| 1-3 | 0.061 | 3.222 | 0.948 | 10.948 |
| 4-6 | 0.571 | 0.766 | 0.304 | 1.929 |
| 7-10 | 0.347 | 1.393 | 0.698 | 2.781 |
| >10 | Ref | -- | -- | -- |
| **Nationality** |  |  |  |  |
| Saudi | **0.014** | 0.234 | 0.074 | 0.742 |
| Non-Saudi | Ref | -- | -- | -- |
| **Location** |  |  |  |  |
| North | 0.381 | 0.710 | 0.331 | 1.526 |
| South | 0.180 | 1.806 | 0.761 | 4.285 |
| East | **0.000** | 5.595 | 2.372 | 13.200 |
| West | 0.745 | 1.143 | 0.510 | 2.561 |
| Central | Ref | -- | -- | -- |
| **Life of Pharmacy Store** |  |  |  |  |
| 1-3 years | **0.001** | 0.228 | 0.096 | 0.539 |
| 4-10 years | 0.435 | 0.784 | 0.426 | 1.444 |
| >10 years | Ref | -- | -- | -- |
| **Weight Mgt Training** |  |  |  |  |
| Yes | **0.028** | 1.992 | 1.075 | 3.688 |
| No | Ref | -- | -- | -- |
| **Weight Mgt Education** |  |  |  |  |
| Yes | 0.100 | 1.763 | 0.897 | 3.465 |
| No | Ref | -- | -- | -- |

*The reference category for practice is “Poor Practice with an average score of <4.21.”

**Supplementary Table 3: Multinomial Regression analysis of factors that are associated with barriers experienced by community pharmacists on weight management.**

| **Characteristics*** | **Significance**  **(*P* value)** | **Odds ratio** | **95% Confidence Interval for Odds ratio** | |
| --- | --- | --- | --- | --- |
|  |  |  | **Lower Bound** | **Upper Bound** |
| **Gender** |  |  |  |  |
| Male | 0.597 | 1.314 | 0.478 | 3.611 |
| Female | Ref | -- | -- | -- |
| **Age (years)** |  |  |  |  |
| 20-30 | **0.037** | 14.607 | 1.174 | 18.789 |
| 31-40 | 0.319 | 3.396 | 0.307 | 17.576 |
| 41-50 | 0.266 | 3.938 | 0.351 | 14.136 |
| >50 | Ref | -- | -- | -- |
| **Education** |  |  |  |  |
| Diploma | **0.000** | 3.951 | 1.229 | 14.225 |
| BSC | **0.000** | 3.669 | 1.142 | 11.631 |
| Pharm.D | **0.000** | 6.326 | 1.119 | 18.663 |
| Postgraduate | Ref | -- | -- | -- |
| **Experience (Years)** |  |  |  |  |
| <1 | 0.195 | 0.286 | 0.043 | 1.903 |
| 1-3 | **0.019** | 0.244 | 0.075 | 0.796 |
| 4-6 | 0.078 | 0.441 | 0.177 | 1.096 |
| 7-10 | 0.382 | 0.743 | 0.382 | 1.446 |
| >10 | Ref | -- | -- | -- |
| **Nationality** |  |  |  |  |
| Saudi | 0.497 | 1.438 | 0.504 | 4.104 |
| Non-Saudi | Ref | -- | -- | -- |
| **Location** |  |  |  |  |
| North | 0.776 | 1.114 | 0.531 | 2.337 |
| South | **0.016** | 0.358 | 0.155 | 0.826 |
| East | **0.001** | 0.252 | 0.113 | 0.561 |
| West | 0.522 | 1.309 | 0.574 | 2.986 |
| Central | Ref | -- | -- | -- |
| **Life of Pharmacy Store** |  |  |  |  |
| 1-3 years | 0.501 | 1.304 | 0.602 | 2.827 |
| 4-10 years | 0.997 | 0.999 | 0.542 | 1.839 |
| >10 years | Ref | -- | -- | -- |
| **Weight Mgt Training** |  |  |  |  |
| Yes | 0.243 | 0.695 | 0.378 | 1.279 |
| No | Ref | -- | -- | -- |
| **Weight Mgt Education** |  |  |  |  |
| Yes | **0.038** | 2.043 | 1.042 | 4.005 |
| No | Ref | -- | -- | -- |

The reference category for the barrier is “High barrier with an average score of ≥3.38.”
